# Study on Qi Deficiency Syndrome Identification Modes of Coronary Heart Disease Based on Metabolomic Biomarkers

**DOI:** 10.1155/2014/281829

**Published:** 2014-03-27

**Authors:** Qi Shi, Huihui Zhao, Jianxin Chen, Youlin Li, Zhongfeng Li, Juan Wang, Wei Wang

**Affiliations:** ^1^The Key Institute of State Administration of Traditional Chinese Medicine, China-Japan Friendship Hospital, Beijing 100029, China; ^2^Beijing University of Chinese Medicine, Beijing 100029, China

## Abstract

Coronary heart disease (CHD) is one of the most important types of heart disease because of its high incidence and mortality. With the era of systems biology bursting into reality, the analysis of the whole biological systems whether they are cells, tissues, organs, or the whole organisms has now become the norm of biological researches. Metabolomics is the branch of science concerned with the quantitative understandings of the metabolite complement of integrated living systems and their dynamic responses to the changes of both endogenous and exogenous factors. The aim of this study is to discuss the characteristics of plasma metabolites in CHD patients and CHD Qi deficiency syndrome patients and explore the composition and concentration changes of the plasma metabolomic biomarkers. The results show that 25 characteristic metabolites related to the CHD patients comparing with the healthy people, and 4 identifiable variables had significant differences between Qi deficiency and non-Qi deficiency patients. On the basis of identifying the different plasma endogenous metabolites between CHD patients and healthy people, we further prompted the metabolic rules, pathogenesis, and biological essence in Qi deficiency syndrome patients.

## 1. Introduction

“Evidence-based medicine” (EBM) has led to a profound change in the practice of the medicine pattern since it came into being for just ten year. The aim of EBM is improving the clinical practice to rigorous standards based on scientific research evidences from the original main basis that personal experience based on. The rapid development of omics technologies made the “bridge” contours between Chinese medicine theory and EBM researches become clear gradually. As a new discipline,omics technology is one of the most active areas in the life sciences. The integrity, dynamic, time and space nature, and complexity of its study features are completely interlinked with traditional Chinese medicine (TCM) theory. The integrity and system of TCM have been reflected at the macrolevel, while omics technology is a reintegration on the global analysis and reduction. Omics technology has provided a new technology platform for the quantitative, objective, standardized, and even EBM study in TCM and its theory.

Syndrome research has always been hot and difficult spots in TCM basic studies. Syndrome differentiation based on information from traditional four diagnostic methods has met challenges and questions with the rapid development and wide application of system biology. The “golden standard” of syndrome diagnosis has not been found yet. The large number and complexity, multilevel relationships of information from four diagnostic methods had constrained the accuracy of syndrome differentiation.

Our earlier study showed that biological parameters could be considered as a reflection of the pathomechanism and physiological mechanism, which might be a reflection of syndrome in TCM too [1]. We have established a mode conducted by four biological parameters, which could distinguish CHD patients with blood stasis syndrome from nonblood stasis syndrome patients by means of C5 Decision Tree [2] and a recognition pattern of CHD patients with Qi deficiency syndrome based on artificial neural network method [3].

For decades, the state has invested a lot of manpower and financial resources for syndrome researches. However, the research has not achieved any breakthrough results and even fallen into the troubles of contradictory and confusion. In recent years, birth of genomics, proteomics, metabolomics, and other modern technology seems to provide a new dawn and bright future for TCM syndrome studies. Omics technology has shown the superiority and advanced nature in the process of TCM syndrome research that no previous technology could match. For instance, we have explored the proteomic specialty and features through seeking the plasma differential proteins in patients with unstable angina of blood stasis pattern and healthy persons by way of two-dimensional difference gel electrophoresis (DIGE) detection [4, 5].

The human body is a complex giant system and syndromes are the overall response of the human body in the pathological conditions. According to the understanding of complex systems, complex system is composed of components (also called subsystems) interacting with each other intricately. The overall behavior of the system cannot be obtained only by the simple act of components (such as the functions or the features). Therefore, research methods and thinking of complex systems should be combined when we study the syndromes, genes, proteins, and metabolomics. In addition, it should be understood that proper research ideas and research methods are usually the key points in syndrome studies [6]. For example, a network balance model can evaluate the imbalanced network underlying TCM syndrome and find potential biomarkers [7]. A systems biology approach with the combination of computational analysis and animal experiment is used to investigate the ZHENG (syndrome) in the context of the neuroendocrine-immune (NEI) system, and the results demonstrate that the ZHENG (syndrome) may have a molecular basis with NEI as background [8].

As one of omics technologies, research methods of metabolomics are distinctive for its overall concept, dialectical concept, and dynamic character, which is very similar to TCM theory. Therefore, metabolomics technology is not only likely to be the communication link between western medicine and TCM, but also the effective tools to reveal the scientific connotation of TCM. TCM syndrome refers to the material bases with the development of itself. These material bases determine the dynamic changes and they are special substance groups when syndromes occurred [9].

The first investigation on the association between traditional tongue diagnosis and the tongue coating microbiome using next-generation sequencing reveals an important connection between the tongue-coating microbiome and traditional tongue diagnosis and illustrate the potential of the tongue-coating microbiome as a novel holistic biomarker for hot or cold syndrome patient [10].

In our early research, the plasma samples of unstable angina (UA) patients and myocardial infarction (MI) swine model with blood stasis syndrome were used to select biomarkers in the level of metabolomics. Twenty-one metabolites in the plasma samples of swine model and 20 metabolites in patients with UA were found to be of significant value. Among which, 8 shared metabolites were found of low level expression in both swine model and UA patients. The results indicated that the 8 shared metabolites can take place of the 21 or 20 metabolites in the classification of swine model with MI and patients with UA, which could be considered as a bridge connecting the mechanism basis and macrosyndromes of swine model with MI and UA patients [11]. Our another metabolomics study on blood stasis syndrome showed glucose metabolism and lipid metabolism disorders reinforce each other, which results a deterioration of coronary artery disease with blood stasis syndrome. These metabolites pattern maybe used as clinical diagnosis and treatment of coronary artery disease indicators [12, 13].

Compared with other metabolomics techniques, the nuclear magnetic resonance (NMR) has many special advantages, such as achievement of noninvasive sample, nonbiased testing, relatively good objectivity and reproducibility, and no need of tedious sample pretreatment. NMR technique can take on a high throughput and lower testing costs. In this study, proton-NMR spectra were collected from human plasma samples of two groups: 15 healthy control and 45 patients with coronary heart disease. The patients were diagnosed and confirmed by coronary angiography. The purpose is firstly to investigate whether we can discriminate healthy control from CHD patients and secondly make a distinction between the patients with Qi deficiency syndrome and non-Qi deficiency based only on the plasma spectral binning data.

## 2. Materials and Methods

### 2.1. Source of Cases

Forty-five cases of CHD in-patients from Anzhen Hospital (Beijing), China-Japan Friendship Hospital (Beijing), and Dongzhimen Hospital affiliated with Beijing University of Chinese Medicine (Beijing) (April 1, 2010, to April 30, 2011). Healthy people cases were 15 volunteers derived from the Hospital of Hubei Medical Center.

### 2.2. Diagnostic Criteria of CHD and TCM Syndrome

All selected patients were diagnosed and confirmed by coronary angiography. Diagnosis criteria of CHD refer to “Treatment guide of stable angina” (ACC/AHA/ACP-ASIM, 1999) and “Diagnosis and treatment recommendations of unstable angina” (Chinese Society of Cardiology, 2000) [14, 15]. Diagnosis criteria of TCM syndrome refer to “Guiding principles for the clinical study of Chinese medicines” (2002) and “Terminology for traditional Chinese medicine clinical practice-part of the syndrome” (1997) [16, 17].

### 2.3. Inclusion, Exclusion, and Rejection Criteria

Inclusion criteria of CHD patients are as follows: (1) aged 20–90 years old, male or female; (2) meeting the unstable angina diagnostic criteria. Inclusion criteria of healthy people cases are as follows: aged 20–90 years old, gender should correspond with the inclusion of patients; examination results were normal. All hospitalized patients had signed informed consent voluntarily.

Excluded cases were patients who suffered from acute myocardial infarction, myocarditis, pericardial disease, cardiac neurosis, intercostal neuralgia, menopausal syndrome, or severe spondylosis; angina caused by rheumatic fever, syphilis, congenital coronary artery abnormalities, hypertrophic cardiomyopathy, aortic stenosis, or regurgitation; stroke, lung infection, nephritis, renal failure, urinary tract infections, rheumatism, severe arrhythmia, heart failure, cancer, and other primary and serious diseases of liver, kidney, and hematopoietic system. Pregnant or lactating women, patients with allergies or psychosis, were also excluded.

*Rejection Criteria.* (1) Violation of inclusion criteria or meeting the exclusion criteria were removed; (2) persons missing the clinical data and who could not be statistically analyzed were removed.

### 2.4. Collections of Clinical Data

General information, history, past medical history, family history, personal history, and signs were collected within 24 hours after the patients were admitted. Details of information from traditional four diagnostic methods were also recorded. Collections of patient histories and information from traditional four diagnostic methods were determined by the relevant professionals. Specific requirements include having the occupation qualification, attending physician or above, and having relevant clinical experience more than two years.

### 2.5. TCM Syndrome Differentiation

TCM syndrome was confirmed by three TCM deputy director physicians who had more than five years of clinical experiences. It should be performed within 24 hours since the patients were admitted to hospital. According to the syndromes differentiation results, the patients were divided into Qi deficiency syndrome and non-Qi deficiency syndrome groups. In 45 patients, 21 cases were Qi deficiency syndrome (47%), while the other 24 patients were with non-Qi deficiency syndrome (53%).

### 2.6. The Main Instruments and Reagent

By CNU VNMRS 600 M Superconducting Fourier NMR Spectrometer (Varian Co., USA), the field strength was superconducting magnet of 14.1 T (600 MHz), equipped with 1H/13C/31P/19F quad cores probe, gradient reversed-phase probe (109Ag-31P), and high resolution solid-state magic angle spinning probe; ultralow temperature freezer (Thermo Revco Value Plus ULT-2586-4-V, the United States of America Thermo Co.); room temperature refrigerator (BCD-215KS, Qingdao Haier Co. Ltd.); small high-speed refrigerated centrifuge (Sigma1-15PK, Germany SIGMA Co.); 98% deuteroxide (D_2_O, Beijing Jingju chemical trade Co. Ltd.).

### 2.7. Collected and Frozen Method of Plasma

Draw elbow vein blood of UA patients and healthy people in the morning after fasting for 12 hours or more. Inject the blood into the anticoagulation blood collection tubes with EDTA-K3. Centrifuge for 20 minutes at 4°C, 3000 r/min. Freeze the supernatant plasma in −80°C refrigerator.

### 2.8. ^1^H-NMR Data Collection

The plasma samples were thawed and centrifuged for 10 min at 4°C, 14000 r/min. 200 *μ*L supernatant was mixed with 400 *μ*L D_2_O and then added to 1.5 mL EP pipe for the second centrifugation under the same conditions above. 550 mL samples supernatant were transferred into the 5 mm NMR tubes. The proton spectra were collected at 25°C on the NMR spectrometer.

The plasma samples data were collected with the methods of Carr-Purcell-Meiboom-Gill (CPMG) and longitudinal eddy delay (LED). CPMG was usually used to observe micromolecule substance, while LED was applied to observe the macromolecule substance. The NMR spectrometer was set. Inhibition of water peak by presaturation, the saturation time was 2 s, mixing time was 0.15 s, the spectrum width was 8012.8 Hz, the sampling points was 32 K, accumulated 64 times, the presaturated and center frequency were both in the water peak position. Free induction decay (FID) signal was transferred into one-dimensional NMR spectra by 32 K Fourier.

Process the spectra with MestReNova software. The concrete steps were as follows: remove the water peak district (4.64/4.78 ppm); adjust the phase and the baseline automatically; take the lactic acid (1.33 ppm) as the chemical shift peak; piecewise integrals were implemented to the spectra within the content of 0.5 ppm to 5.5 ppm (CPMG test and LED test) using a 0.04 ppm bin width. Further analysis was carried out after the integral value of each spectrum was normalized intensively and scaled by median method.

### 2.9. ^1^H-NMR Data Analysis Method

Further analysis was carried out after the integral value of each spectrum was normalized intensively and scaled by median method. First of all, take the OPLS-DA analysis by SIMCA-P software (Umetrics, Umea, Sweden). Secondly, according to the results of VIP value, the normalized integral values of the main plasma metabolites from the CHD groups and the healthy people control groups were conducted with statistical analysis of *t*-test/nonparameter test method. The statistically difference was significant with the *P* value that was less than 0.05, and extremely significant difference was showed when the *P* value was less than 0.01. In order to screen the different metabolites between Qi deficiency and non-Qi deficiency groups, we applied principal component analysis (PCA), partial least squares regression analysis (PLS-DA), and hierarchical clustering to comprehend inner rules of variables in the data. These methods were performed with software “MetaboAnalyst 2.0” (online website: http://www.metaboanalyst.ca/MetaboAnalyst/faces/Home.jsp)

## 3. Results

### 3.1. Demographic Details of CHD Patients and Healthy People

There was no difference in the age and the gender ratio between the UA patients groups and the control group (*P* > 0.05) (see Table 1). There was no difference in the aspect of age, gender, body mass index, the course of CHD, diabetes combination, hyperlipemia combination, use of antiplatelet drugs, nitrate esters drugs, statins, ACEI/ARB, beta blocker, and calcium channel antagonist between the patients with or without Qi deficiency syndrome (*P* > 0.05) (see Table 2).

### 3.2. ^1^H-NMR Metabolic Fingerprinting and Identification Results in CHD Patients and Healthy People

**Figure 1:**
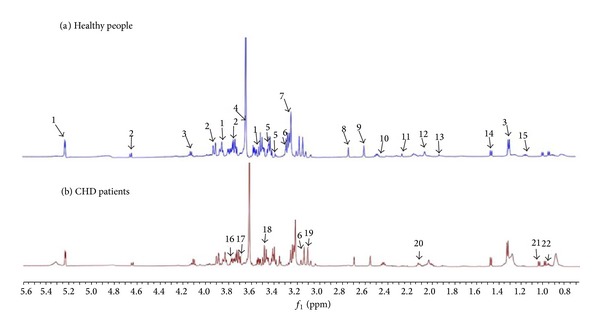
CGMP ^1^H-NMR spectra of CHD patients and healthy people.* Note*. (a) Healthy people; (b) CHD patients: 1: *α*-glucose, 2: *β*-glucose, 3: lactic acid, 4: creatine, 5: praline, 6: histidine, 7: choline, 8: aspartic acid, 9: methylamine, 10: carnitine, 11: acetone, 12: acetyl glutamic acid, 13: lysine, 14: alanine, 15: *β*-hydroxyisobutyric acid, 16: glutamine, 17: isoleucine, 18: tryptophan, 19: phenylalanine, 20: glutamate methylamine, 21: valine, and 22: leucine.

**Figure 2:**
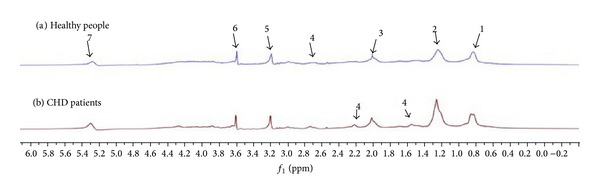
LED ^1^H-NMR spectra of CHD patients and healthy people.* Note*. (a) Healthy people; (b) CHD patients: 1: low density lipoprotein/very low density lipoprotein, 2: high density lipoprotein, 3: N-acetyl glycoprotein, 4: lipid, 5: choline, 6: lipid compound, and 7: unsaturated fatty acids.

Thirty-nine endogenous metabolites had been detected through the identification, of which 34 were micromolecule and 5 were macromolecule. The micromolecule substances were *α*-glucose, *β*-glucose, *β*-hydroxyisobutyric acid, *β*-hydroxybutyric acid, phenylalanine, alanine, acetone, choline, methionine, dimethylamine, glycine, glutamate methylamine, glutamine, creatine, creatinine, inositol, methylamine, lysine, leucine, tyrosine, hippuric acid, ornithine, taurine, praline, carnitine, lactic acid, tryptophan, threonine, aspartic acid, valine, isoleucine, acetyl glutamic acid, histidine, and N-acetyl glycoprotein. The macromolecule substances were unsaturated fatty acids, lipid compound, lipid, low density lipoprotein/very low density lipoprotein, and high density lipoprotein (see Figures 1 and 2 and Table 3).

### 3.3. ^1^H-NMR Recognition Pattern Results between CHD Patients and Healthy People

**Figure 3:**
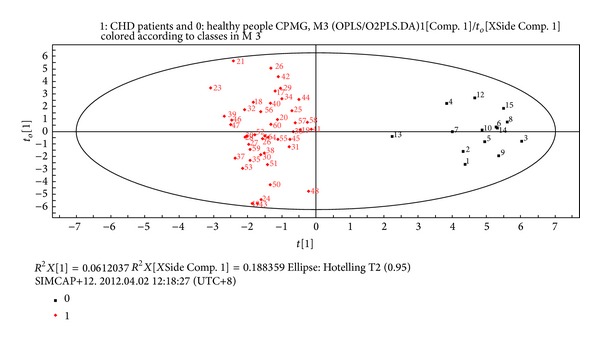
Plasma CPMG metabolites OPLS/O2PLS-DA integral matrix figure in CHD patients and healthy people groups.* Note*. 0: healthy people group; 1: CHD patients group.

**Figure 4:**
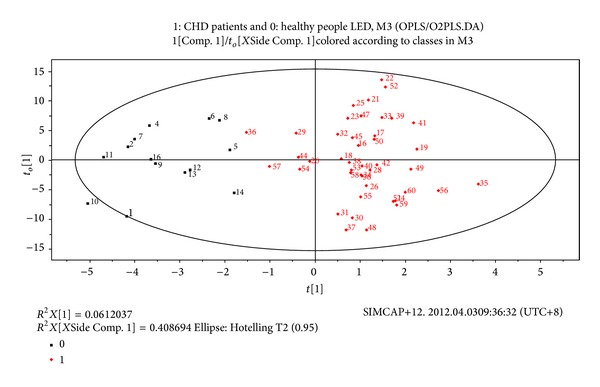
Plasma LED metabolites OPLS/O2PLS-DA integral matrix figure in CHD patients and healthy people groups.* Note*. 0: healthy people group; 1: CHD patients group.

OPLS/O2PLS-DA integral matrix figures results of CPMG test and LED test showed distribution region of CHD patients and healthy people could be completely separated along the *t*(1) axis direction. The fitting degree of the modes was well, and there were no special nodes, cross or overlapping nodes (see Figures 3 and 4).

According to the VIP figures of CPMG and LED detection results, we selected 44 identifiable variables with the VIP value higher than 1. These variables have lager contributions to the classification because the locations of these variables were far from the origin in the corresponding load and S figures. At the same time, these variables had a good correlation with the main composition 1. *t*-Test/nonparametric test was applied to these variables, and compared with the healthy people, 37 identifiable variables had significant differences (*P* < 0.05 or *P* < 0.01). Finally, we obtained 25 characteristic metabolites related to the CHD patients after the integration of the 37 variables. The 25 characteristic metabolites were listed in Table 4. Among these 25 characteristic metabolites, *β*-hydroxyisobutyric acid of the CHD patients was downregulated, while the other 24 characteristic metabolites were upregulated compared with the healthy control group.

### 3.4. ^1^H-NMR Recognition Pattern Results between Qi Deficiency and Non-Qi Deficiency CHD Patients

#### 3.4.1. Result of PCA and PLS-Da Analysis

**Figure 5:**
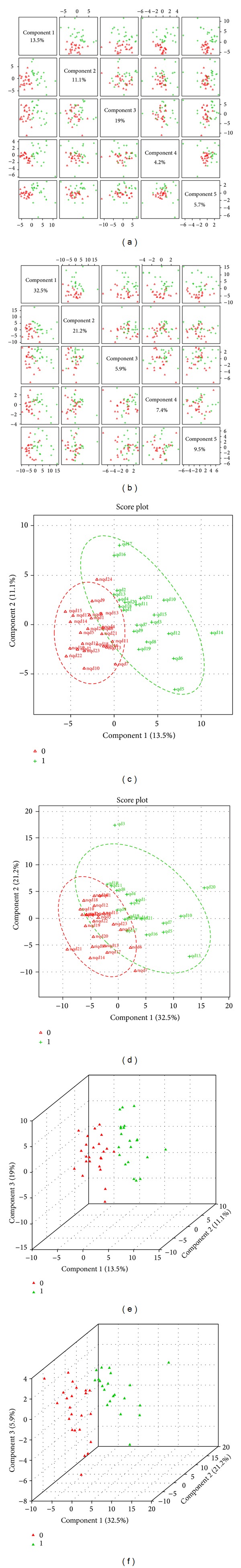
PCA and PLS-DA scores in Qi deficiency and non-Qi deficiency groups.* Note*. (a) The top five PCs overview for CPMG; (b) the five PCs overview for LED; (c) and (e) 2D and 3D score plot of PLS-DA for CPMG; (d) and (f) 2D and 3D score plot of PLS-DA for LED. In (c) and (d), nqd = non-Qi deficiency group, qd = Qi deficiency group. In (e) and (f), Red triangle = non-Qi deficiency group; green triangle = Qi deficiency group. For CPMG,* R* ^2^ *X* = 0.42634,* R* ^2^ *Y* = 0.86034, and* Q* ^2^ = 0.88. For LED,* R* ^2^ *X* = 0.38967,* R* ^2^ *Y* = 0.8136, and* Q* ^2^ = 0.9375.

Principal component analysis (PCA) provides an excellent visualization tool of high-dimensional data by projecting the data into low-dimensional space (usually 2D or 3D). Figures 5(a) and 5(b) were the PCA analysis results overview of pairwise score plots from the top five PCs.

PLS-DA was used to maximize the difference of metabolic profiles between Qi deficiency and non-Qi deficiency groups and facilitate the detection of metabolites consistently present in the plasma samples. In Figures 5(c), 5(d), 5(e), and 5(f), distribution region of the two groups could be completely separated and there were no special nodes.

#### 3.4.2. Result of VIP Value of Each Variable

**Figure 6:**
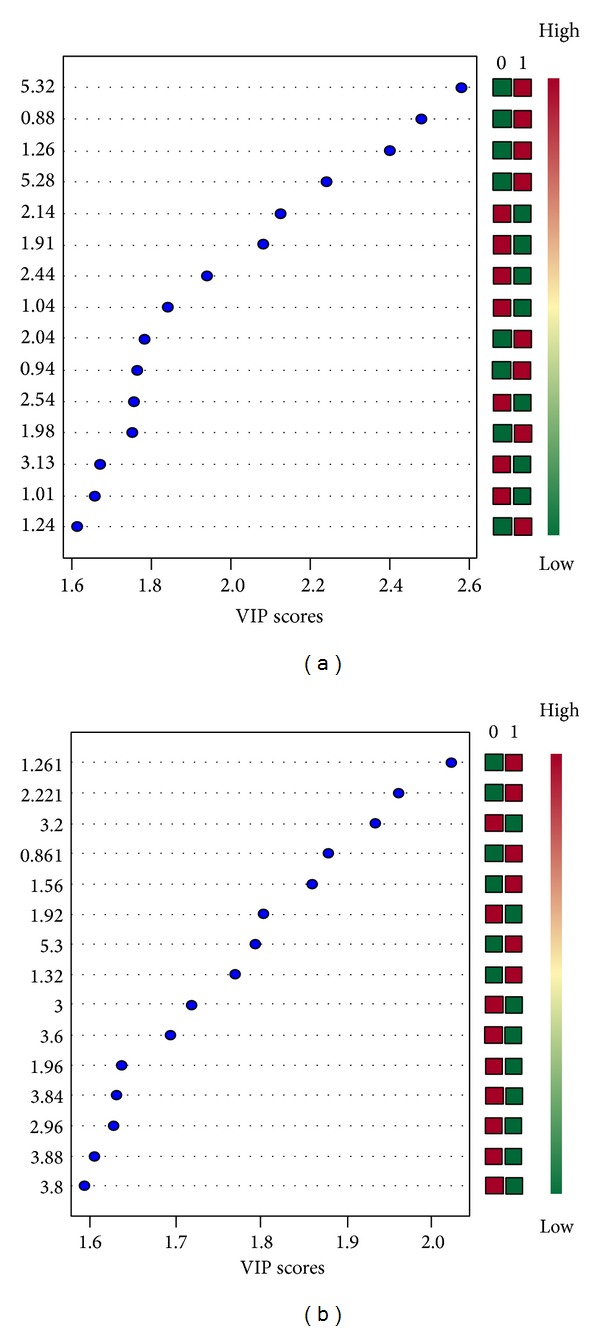
The VIP value of the variables.* Note*. (a) The top 15 significant features of the metabolite markers based on the VIP scores for CPMG; (b) the top 15 significant features of the metabolite markers based on the VIP scores for LED.

According to the VIP results, Figures 6(a) and 6(b) separately showed the top 15 significant features of the metabolite markers based on the VIP projection for CPMG and LED. In CPMG results, the decreasing order of the VIP values was acetyl glutamic acid (*δ*1.26), glutamine (*δ*2.14), lysine (*δ*1.91), carnitine (*δ*2.44), valine (*δ*1.04), isoleucine (*δ*1.98), acetyl glutamic acid (*δ*0.94), methylamine (*δ*2.54), acetyl glutamic acid (*δ*1.98), phenylalanine (*δ*3.13), and acetyl glutamic acid (*δ*1.01). In LED results, the decreasing order of the VIP values were lipid (*δ*1.56), lipid compound (*δ*3.60), and *α*-glucose (*δ*3.84).

#### 3.4.3. Result of Hierarchical Clustering

**Figure 7:**
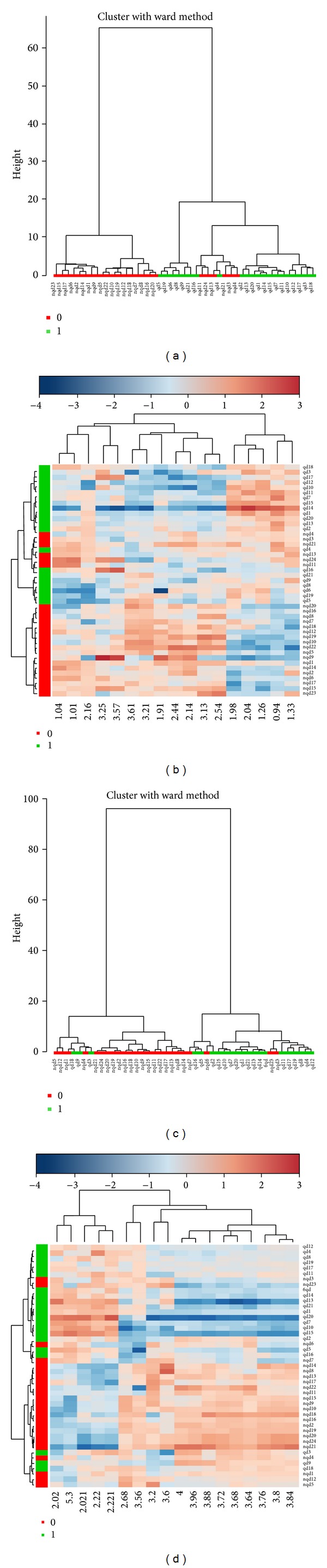
The dendrogram and heatmap of hierarchical clustering for CPMG and LED.* Note*. (a) The dendrogram for CPMG; (b) the heatmap for CPMG; (c) the dendrogram for LED; (b) the heatmap for LED. 0: non-Qi deficiency group, 1: Qi deficiency group.

Hierarchical clustering is commonly used for unsupervised clustering. Agglomerative hierarchical clustering begins with each sample as separate cluster and then proceeds to combine them until all samples belong to one cluster. The result is usually presented as a dendrogram or heatmap. The results showed that CHD patients with Qi deficiency syndrome or non-Qi deficiency syndrome could be distinguished well (see Figure 7).

#### 3.4.4. Plasma ^1^H-NMR Biomarkers in CHD Qi Deficiency Patients

According to the VIP figures of CPMG and LED detection results, we selected identifiable variables with the VIP value higher than 1. *t*-Test/nonparametric test was applied to these variables, and compared with the non-Qi deficiency patients, 4 identifiable variables had significant differences (*P* < 0.05) (see Table 5).

## 4. Discussion

Compared with transcriptomics and proteomics, metabonomics has obvious advantages. Firstly, small changes in gene and protein expression will be amplified in metabolite, which make the detection much easier; secondly, in metabonomics, it is not necessary to form the whole genome sequencing or the database for a large sequence tags expressions; thirdly, the number of metabolites varieties is far less than that of the gene and protein; and fourthly, the study technology is in common use for the tissue metabolites which are similar [18]. Correlation studies on metabonomics and CHD have been reported many times. For instance, in the pathogenesis of CHD, Mayr et al. [19] have combined the applications of proteomics and metabolomics technology to study the vascular smooth muscle in mice. In their research, the cell protein changes have been linked to their function changes through the two methods, and it reveals the formation mechanism of atherosclerosis from multiangle. In predicting the severity of coronary artery disease [20], subjects were divided into two groups: three branches lesion group and normal control group. Brindle had detected the serum samples from the two groups by the means of ^1^H NMR. In the results, the PLS-DA mathematical model could predict the three branch lesions. The sensitivity and specificity could reach 92% and 93%. In a clinical research [21], serum samples from 86 CHD patients confirmed by coronary angiography and 20 healthy volunteers were detected with ^1^H-NMR. The results showed that unsaturated fatty acid, lactic acid, alanine, glutamate, glucose, lipid, low density lipoprotein/very low density lipoprotein, betaine, phosphocholine, taurine, choline, phosphatidyl choline, and high density lipoprotein could be served as metabolic biomarkers of CHD patients. The occurrence of CHD angina pectoris closely is associated with energy metabolism, lipid metabolism, and glucose metabolism.

In our study, we obtained 25 characteristic metabolites related to CHD patients by comparing the plasma metabolites between CHD patients and healthy people. The 25 characteristic metabolites were listed in Table 4 and had participated jointly the following metabolic process in summary.

(1) Metabolic processes of the amino acids: a research showed that the levels of 20 amino acid in CHD patients were significantly higher that healthy people and 7 kinds of essential amino acid increased much more obviously [22]. Dr. Luo has detected the free amino acid in serum from 30 AMI patients, and the results were compared with 60 healthy people and 28 CHD cases. In AMI cases, the levels of threonine, serine, glutamic acid, alanine, cystine, valine,leucine, tyrosine, phenylalanine, lysine, histidine, and proline were higher than that in healthy people. The content of alanine, isoleucine, leucine, tyrosine, and phenylalanine in AMI patients was obviously higher than that in CHD patients [23]. In our study, plasma leucine, phenylalanine, lysine, glutamic acid, glutamine, tyrosine, tryptophan, ornithine, proline, threonine, aspartic acid, valine, isoleucine, acetyl glutamic acid, and histidine of the CHD patients had increased significantly. On one hand, these results showed that the majority of CHD patients are in the state of over nutrition. The surplus amino acids provided adequate raw materials for the synthesis of lipid; thus the situation of abnormal lipid metabolism of UAP patients had been aggravated at the same time. On the other hand, free radical lipid peroxidation in UAP patients is usually active. In this process, the decomposition hormone increased, and the energy consumption accelerated, which then caused the lack of the whole body energy and the increases in individual amino acids in plasma.

(2) Metabolic processes of glucose: abnormal glucose metabolism often dues to the increase level of AGEs in vivo, which resulted from the increase of endogenous synthesis, exogenous uptake, and decrease of catabolism. By ways of protein and lipoprotein glycosylation, formation of collagen cross-linking, and subendothelial matrix saccharification, the AGEs inhibit the nonreceptor dependent pathway and receptor dependent pathways and then accelerate the process of atherosclerosis. Our research found that the level of N-acetyl glycoprotein, *α*-glucose, and *β*-glucose in the plasma of AHD patients was higher than that in healthy people. It proved that significant disorders of glucose metabolism were happened in CHD patients.

(3) Metabolic processes of lipids: lipid disorder has been associated with the pathogenesis of CHD. The increased plasma lipid composition corrodes the arterial walls, result in proliferation of vascular smooth muscle cells. With the blood lipids infiltrating foam cells, proteoglycan stimulates the fibrous tissue proliferation continuously, then the AS plaque formation. In the hyperlipidemic state, lipid metabolism in the vascular cells replaces the sugar metabolism, which decreases the vessels main function material significantly; when the excess fatty acids enter the mitochondria, crisis situations of oxidation-phosphorylation uncoupling will happen and finally lead to energy metabolite depletion, further mitochondrial dysfunction, and the AS occurrence ultimately [24]. Our study also confirmed that there were abnormalities of lipid metabolism in CHD patients and mainly in the form of increases of the plasma lipid contents. However, the results also showed that the level of HDL in CHD patients was also higher than that in healthy people. We considered that the intervention of statins drugs led to these results.

(4) Metabolic processes of energy: lactic acid is the intermediate products of in vivo three-tricarboxylic acid cycle, and abnormal metabolism of the lactic acid is the mark of energy metabolism abnormality. Myocardial oxygen intake was decreased when myocardial ischemia exists. In the situation of serious myocardial hypoxia, the glucose could not enter the cycle of three-tricarboxylic acid after its glycolysis into pyruvic acid and then reduced into lactic acid. Serious accumulation of the lactic acid can cause the systemic acidosis. Intracellular excessive hydrogen ion inhibit the binding between calcium ion and myocardial contractile protein. Decreases of the myocardial contractility, declines of the effective circulation, and slower oxygen exchanges between tissues and blood have aggravated the degree of the acidosis. Studies had suggested that the accumulation of acidic products in the myocardial cells was the main causes of cardiac pains [25, 26]. In this study, the plasma lactic acid in CHD patients increased obviously. It suggested the abnormal metabolic processes of energy and acidosis situations in the patients.

(5) Coagulation progress: as we all know that the fibrinogen, prothrombin, and several clotting factors participating in process of the blood clotting are all glycoproteins [27], in our results increases of the N-acetyl glycoprotein suggested the abnormal appearances of the clotting mechanism in CHD patients.

(6) Other metabolic processes: metabolic abnormalities of the alanine were always connected with the disorders of the glomerular filtration and recovery functions. Methylamine is exogenous or endogenous amines. Current studies have shown that chronic exposure of the methylamine could accelerate the generations of many metabolites through catalysis of semicarbazide-sensitive amine oxidase. The products usually included formaldehyde, H_2_O_2_, and ammonia, which could lead to the endothelial injury [28]. The plasma alanine and methylamine contents are elevated in our research in the UA patients, and it prompted the possibilities of the renal function impairment and the vascular endothelium injury in CHD patients.

Synthesizing our analytic results of PCA and PLS-DA scores, VIP value of the variables, and *t*-test/nonparametric test, we selected the identifiable metabolites biomarker related to Qi deficiency CHD patients. They were acetyl glutamic acid, lysine, valine, and carnitine. The four metabolites were all in a downward trend in Qi deficiency patients.

Lysine is one of the essential amino acids. Lysine deficiency will lead to a series of body symptoms, including the body fatigue, weakness, nausea, vomiting, dizziness, loss of appetite, and growth retardation. Clinical studies have shown that lysine can increase the SOD and CAT activities in blood, decrease the oxygen free radicals content, and maintain normal physiological functions for brain cells, and muscle tissues provide the essential amino acid and energy sources for repair of damaged tissue, which is very useful for enhancing the ability of sports [29]. Dr. Luo explored the influence of different concentration lysine supplementation on the heart and liver cell apoptosis in exhaustive-exercise rats. Their results found that regulatory gene Bax and Bcl-2 optical density values of heart and liver cells apoptosis had significant difference among the control, exercise, and drug intervention groups [30]. Our study suggested that the plasma lysine expression decreased obviously in CHD Qi deficiency patients. Low lysine level is not conducive to free radical scavenging in vivo. The effect of inhibitory cell apoptosis has been weakened, and it finally results in the incidence of AS. At the same time, lysine deficiency may be one of the reasons that shortness of breath and fatigue are prone to appear in CHD patients with Qi deficiency syndrome.

Carnitine is a kind of carrier transport. The main function of carnitine is to transport long chain fatty acids across the inner mitochondrial membrane into the mitochondrial matrix for *β*-oxidation, energy supply, and promotion fat burning [31]. Carnitine deficiency will lead to fat metabolism disorder and affect the energy supply, which causes many kinds of diseases. Carnitine is transported into the organization through blood circulation, mainly distributed in the cardiac and skeletal muscle [32]. Study has confirmed that plasma free carnitine level in diabetes or CHD patients was significantly decreased than that in healthy people. The situation was much serious in the patients who have diabetes and CHD [33]. In this study, plasma carnitine in CHD patients with Qi deficiency syndrome was lower significantly than that in non-Qi deficiency patients. Qi deficiency patients also have the abnormalities in the glucose and fat utilization, including increased *α*-glucose and *β*-glucose and decreased plasma lipid and HDL. In the abnormal process, fat synthesis in the liver reduced, catabolism increased, *β*-oxidation enhanced, carnitine in plasma, and muscle flowed to the liver to participate in the *β*-oxidation, leading to increased consumption of carnitine.

In addition, patients with Qi deficiency syndrome have given expression to disturbance of amino acid metabolism: decrease in acetyl glutamic acid, valine, glutamine, proline, and aspartic acid and increase in tryptophan, histidine, tyrosine, isoleucine, glycine, glutamate methylamine, ornithine, and taurine. Abnormalities metabolism of glutamate methylamine was associated with central fatigue [34]. Dizziness and fatigue symptoms in Qi deficiency syndrome patients may be associated with glutamate metabolism dysfunction. Significantly reduced levels in glutamine in Qi deficiency patients had a certain correlation with immune function decline [35]. Taurine, also called *α*-amino acetic acid, firstly separated from the bezoar, is a nonprotein amino acid containing sulfur. It is in a free state in vivo and not involved in protein biosynthesis. Taurine can promote the brain tissue and mental development in the infant, improve nerve conduction and visual function, prevent cardiovascular disease, improve endocrine condition, and enhance the immunity of the human body. Taurine played a protective role in the dysfunction of myocardial nuclear calcium transport when the myocardial injury occurred in rats [36]. Dr. Sun et al. found that myocardial cells apoptosis exits in the rat heart during hypothermic preservation, while the apoptosis had been reduced using the taurine as preserve solution. Taurine levels in the patients with Qi deficiency may prompt the start of body protection function [37].

## 5. Conclusion

In summary, the plasma metabolic characteristics in CHD patients or CHD Qi deficiency patients were both related to metabolic pathways of energy metabolism, amino acid metabolism, glucose metabolism, lipid metabolism, and oxidative stress. Synthesis results of PCA, PLS-DA, VIP, and *t*-test/nonparameter test prompted the characteristic metabolic biomarkers in CHD and CHD Qi deficiency patients. These biomarkers pointed out that the CHD pathogenesis also could reflect the characteristic in common or respective characteristic for Qi deficiency syndrome to a certain extent. According to the results of hierarchical clustering, the characteristics metabolites could basically achieve the distinction between Qi deficiency and non-Qi deficiency.

## Figures and Tables

**Table 1:** Demographic details of CHD patients and healthy people.

	CHD group (*n* = 45)	Healthy group (*n* = 15)	*P* value
Age	59.24 ± 7.87	57.53 ± 4.47	0.304
Gender (male/female)	33/12	7/8	0.058

**Table 2:** Demographic details of CHD patients with or without Qi deficiency syndrome.

	Qi deficiency (*n* = 21)	Non-Qi deficiency (*n* = 24)	*P* value
Age	59.76 ± 8.61	58.79 ± 7.32	0.685
Gender (male/female)	14/7	19/5	0.344
BMI	25.57 ± 2.49	26.77 ± 6.17	0.750
Years of CHD	3.71 ± 4.16	3.00 ± 3.24	0.419
Diabetes combination	13 (61.9%)	17 (70.8%)	0.526
Hyperlipemia combination	6 (28.6%)	4 (16.7%)	0.338
Use of antiplatelet drugs	21 (100%)	24 (100%)	/
Use of nitrate esters drugs	11 (52.4%)	13 (54.2%)	0.905
Use of statins	14 (66.7%)	19 (79.2%)	0.344
Use of ACEI/ARB	2 (9.5%)	2 (8.3%)	0.889
Use of beta blocker	12 (57.1%)	16 (66.7%)	0.511
Use of calcium channel antagonist	21 (100%)	24 (100%)	/

**Table 3:** Plasma metabolite identification in CHD patients and healthy people.

Metabolite	Attribution	*δ* (^1^H)	Multiplicity
*α*-Glucose	CH_1_, C–H_7_, C–H_2_	5.23, 3.84, 3.53	d, m, dd
*β*-Glucose	CH_1_, C–H_6_, C–H_6_′	4.64, 3.90, 3.72	d, dd, dd
*β*-Hydroxybutyric acid	CH_2_	2.31	abx
*β*-Hydroxy isobutyric acid	CH_3_	1.20	d
Phenylalanine	*α*-CH, *β*-CH_2_	4.00, 3.13	m, m
Alanine	CH_3_	1.48	d
Acetone	CH_3_	2.23	s
Choline	N(CH_3_)_3_, OCH_2_	3.21, 4.07	s, m
Methionine	*β*-CH_2_	2.16	m
Dimethylamine	CH_3_	2.72	s
Glycine	CH_2_	3.57	d
Glutamate methylamine	*β*-CH_2_	2.10	m
Glutamine	*α*-CH, *γ*-CH_2_, *β*-CH_2_	3.77, 2.46, 2.14	t, m, m
Creatine	CH_2_	3.61	s
Inositol	CH_5_	3.28	t
Creatinine	CH_2_	4.06	s
Methylamine	CH_3_	2.54	s
Lysine	*ε*-CH_2_, *β*-CH_2_, *δ*-CH_2_	3.03, 1.91, 1.73	t, m, m
Tyrosine	CH, CH_2_	3.94, 3.06	abx, abx
Leucine	*δ*-CH_3_	0.97	t
Hippuric acid	CH_2_	3.97	d
Ornithine	*α*-CH	3.79	t
Taurine	NCH_2_	3.43	t
Praline	*δ*-CH_2_, *δ*-CH_2_, *α*-CH_2_	3.42, 3.33, 2.35	m, m, m
Carnitine	CH_2_(COO)	2.44	dd
Lactic acid	CH, CH_3_	4.12, 1.33	q, d
Tryptophan	CH_2_, CH_2_	3.49, 3.31	abx, abx
Threonine	CH_3_	1.34	d
Aspartic acid	*β*-CH_2_	2.68	abx
Valine	*β*-CH, *α*-CH, CH_3_	2.28, 3.62, 1.04	m, d, d
Isoleucine	*β*-CH_2_, CH_3_	1.89, 2.04	m, s
Acetyl glutamic acid	*β*-CH, *γ*-CH_2_, *β*-CH_3_, *δ*-CH_3_, *α*-CH	1.98, 1.26, 1.01, 0.94, 3.68	m, m, d, t, d
Histidine	*β*-CH_2_, *β*-CH_2_	3.25, 3.14	abx, abx
High density lipoprotein	–CH_3_	1.26	m
Low density lipoprotein/very Low density lipoprotein	–(CH_2_)_*n*_–	0.86/0.9	m
N-Acetyl glycoprotein	–CH_2_CH=	2.02	m
Unsaturated fatty acids	–CH=CH–	5.26	m
Lipid compound	/	3.60	m
Lipid	=CHCH_2_CH=, –CH_2_CO, –CH_2_CH_2_CO	2.74, 2.22, 1.61, 1.56, 2.68	m, m, m, m, m

*Note*. s: unimodal; d: doublet; t: triplet; q: quartet; m: multiplet; abx: the secondary coupling; dd: double doublet.

**Table 4:** The characteristics metabolites related to CHD patients.

Metabolites	Chamical shift	*t*/*z* value	*P* value	Upregulation/downregulation
Leucine	0.97	−3.867	0.000	↑
N-Acetyl glycoprotein	2.02	−2.552	0.011	↑
*α*-Glucose	3.53, 5.23, 3.84	−3.576/−3.372/−3.116	0.000/0.001/0.002	↑
*β*-Glucose	4.64, 3.72, 3.9	−3.081/−3.372/−3.491	0.002/0.001/0.000	↑
*β*-Hydroxy isobutyric acid	1.2	2.082	0.042	↓
Phenylalanine	3.13	−3.322	0.002	↑
Acetone	2.23	−2.860	0.006	↑
HDL	1.26	−2.296	0.022	↑
Glutamate	2.1	−3.030	0.002	↑
Glutamine	2.14, 3.77	−2.761/−2.603	0.008/0.009	↑
Methylamine	2.54	−2.422	0.019	↑
Lysine	1.73	−2.194	0.028	↑
Tyrosine	3.94	−2.074	0.038	↑
Ornithine	3.79	−2.467	0.014	↑
Taurine	3.43	−3.918	0.000	↑
Proline	3.42	−3.747	0.000	↑
Lactic acid	1.33	−2.842	0.004	↑
Tryptophan	3.49	−3.628	0.000	↑
Threonine	1.34	−2.057	0.040	↑
Aspartic acid	2.68	−2.339	0.023	↑
Valine	1.04, 2.28	−3.783/−2.738	0.000/0.008	↑
Acetyl glutamic acid	2.04	−3.047	0.002	↑
Isoleucine	1.01, 1.98, 0.94, 1.26	−2.851/−2.731/−2.228/−2.330	0.006/0.006/0.026/0.020	↑
Lipids	2.22	−2.262	0.024	↑
Histidine	3.25	−3.047	0.002	↑

*Note*. Up- or downregulation mean CHD patients group comparing with healthy people group.

**Table 5:** The characteristics metabolites related to CHD Qi deficiency patients.

Metabolites	Chamical shift	*t*/*z* value	*P* value	Upregulation/downregulation
Acetyl glutamic acid	1.89	2.182	0.035	↓
Lysine	1.91	−2.457	0.014	↓
Valine	2.28	2.167	0.036	↓
Carnitine	2.44	2.273	0.028	↓

*Note*. Up- or downregulation mean Qi deficiency patients group comparing with non-Qi deficiency patients group.
